# The prognostic utility of the ratio of lymphocyte to monocyte in patients with metastatic colorectal cancer: a systematic review and meta-analysis

**DOI:** 10.3389/fonc.2025.1394154

**Published:** 2025-02-03

**Authors:** Pingping Mei, Li Huang, Lu Lin, Yan Chen, Xiutian Guo

**Affiliations:** ^1^ Shanghai Municipal Hospital of Traditional Chinese Medicine, Shanghai University of Traditional Chinese Medicine, Shanghai, China; ^2^ Anorectal Department, Affiliated Hospital of Shaanxi University of Chinese Medicine, Xianyang, Shaanxi, China

**Keywords:** lymphocyte-to-monocyte ratio, metastatic colorectal cancer, survival, prognosis, meta-analysis

## Abstract

**Background:**

Although advancements in treatment have resulted in improved overall outcomes for patients diagnosed with colorectal cancer, the prognosis remains unfavorable for individuals with metastatic colorectal cancer (mCRC). The association between lymphocyte/monocyte ratio (LMR) and clinical outcomes in mCRC patients is a subject of controversy. To systematically evaluate the correlation between LMR and prognostic factors in individuals with mCRC, we conducted this meta-analysis.

**Methods:**

The databases PubMed, Embase, Web of Science, and the Cochrane Library were systematically searched for all relevant studies from their inception until October 26, 2024. Study selection was conducted based on predetermined inclusion and exclusion criteria. The primary outcomes of interest included prognosis measures such as overall survival (OS), progression-free survival (PFS), disease-free survival (DFS), and cancer-specific survival (CSS) in patients with metastatic colorectal cancer. Random-effects models or fixed-effects models were used to determine the pooled risk ratio (HR) and corresponding 95% confidence interval (CI) for each outcome indicator. Additionally, the pooled odds ratio (OR) and its corresponding 95% CI were calculated for LMR and clinicopathological characteristics.

**Results:**

Fourteen studies involving 3,089 patients were included in the analysis. The pooled analysis found that high LMR was correlated with better OS (HR: 0.55, 95% CI: 0.49-0.62, p<0.00001), PFS (HR: 0.68, 95% CI: 0.57-0.81, p<0.0001) and CSS(HR: 0.55, 95% CI: 0.32-0.95, p=0.03),The prognostic value of high LMR values for DFS(HR: 0.93, 95% CI: 0.78-1.12, p=0.46) in patients with metastatic rectal cancer was not found to be significant. We performed subgroup analyses based on study characteristics to confirm the robustness of our findings. Further clinicopathological analysis showed no significant difference between patients with elevated LMR and those without elevated LMR.

**Conclusions:**

In conclusion, the results demonstrate a robust correlation between elevated LMR levels and a favorable prognosis in terms of overall survival (OS), progression-free survival (PFS), and cancer-specific survival (CSS) among patients diagnosed with metastatic colorectal cancer. However, further high-quality prospective studies are warranted to validate our findings since the majority of current investigations have relied on retrospective study designs.

**Systematic review registration:**

https://www.crd.york.ac.uk/prospero/display_record.php?ID=CRD42024496467, identifier CRD42024496467.

## 1 Introduction

Colorectal cancer (CRC) ranks as the fourth most common cause of cancer-related deaths worldwide, resulting in nearly 900,000 fatalities annually (1). Metastasis is initially observed in approximately 20% to 30% of patients upon colorectal cancer diagnosis, and about 10% to 25% of patients develop metachronous metastasis following treatment-oriented surgery (2). Metastatic colorectal cancer (mCRC) carries a grim prognosis, with a 5-year survival rate of less than 20% (3). Many factors can predict the prognosis of colorectal cancer, such as tumor stage, cell differentiation, vascular invasion, nerve invasion, etc. However, some patients with good prognostic factors still have poor prognosis. Therefore, finding other novel biomarkers to predict the prognosis of colorectal cancer and help select the best treatment strategy remains a challenge in current clinical practice.

It is increasingly acknowledged that tumor occurrence is not solely determined by tumor-related factors but also significantly influenced by the immune status of the host (4–6). Recent studies have demonstrated that systemic inflammation facilitates tumor metastasis through various mechanisms (7). Therefore, the inflammatory response within the host system plays a pivotal role in tumor initiation, progression, malignant transformation, invasion, and metastasis. Alterations in peripheral blood cell composition including lymphocytes, monocytes, neutrophils, and platelets can be used to assess systemic inflammation. Parameters such as neutrophil-to-lymphocyte ratio (NLR), platelet-to-lymphocyte ratio (PLR), and systemic immune inflammation index (SII) are employed to characterize these changes (8). A large number of studies have shown that these biomarkers play a prognostic role in different tumors such as lung cancer, colorectal cancer, kidney cancer, and melanoma; higher NLR and PLR indicate worse prognosis (9–11). In recent years, there have been proposals to use the combination of lymphocytes and monocytes for measuring the host systemic inflammatory response, specifically the lymphocyte-to-monocyte ratio (LMR). The LMR, which can be easily calculated from a complete blood count of peripheral blood, has been reported as a novel prognostic indicator based on inflammation in recent years. Studies have shown that an increase in LMR is closely associated with a favorable prognosis in patients with pancreatic cancer, lung cancer, liver cancer, ovarian cancer, and breast cancer (12), although some authors disagree due to differences in study design and sample size (13–16). A previous meta-analysis conducted by Hamid et al. (17) reported that LMR was superior to PLR as a predictor of long-term prognosis in colorectal patients.

Furthermore, previous studies have demonstrated that a low-preconditioning lymphocyte-to-monocyte ratio (LMR) is an important prognostic biomarker for poor survival in rectal cancer patients who undergo radical resection or chemotherapy (18, 19). To the best of our knowledge, there are currently no systematic reviews and meta-analyses establishing an association between LMR and survival in metastatic colorectal cancer (mCRC) patients. Therefore, we conducted this meta-analysis to investigate the prognostic significance of LMR in patients diagnosed with mCRC, aiming to provide robust evidence-based medical support and objective data for clinical decision-makers.

## 2 Materials and methods

### 2.1 Literature search

This systematic review study process followed the Preferred Reporting Items for Systematic Reviews and Meta-Analyses (PRISMA 2020) statement (20), and the protocol was registered with the International Prospective Registry of Systematic Reviews (PROSPERO: CRD42024496467). Two investigators, the MPP and LL, were responsible for developing the search strategy. We conducted a systematic literature search at PubMed, Cochrane Library, Embase, and Web of Science for eligible studies from database construction until October 26, 2024. The search strategy is based on the following keywords: colorectal tumor, “intestine tumor”, “cecum tumor”, “colon tumor”, “Colorectal tumor” Neoplasms, prognosis, survival, Prognostic Factors, outcome, Lymphocytes, Monocytes, lymphocyte ratio(MLR) and lymphocyte monocyte ratio(LMR), detailed search strategies are shown in **Supplementary Table S1**. Additionally, the two researchers independently screened titles and abstracts, obtained full-text articles, and evaluated them to identify qualified studies. Any disagreements in the literature search were resolved through consensus.

### 2.2 Study selection

The inclusion criteria are as follows:(1) The diagnosis of mCRC was made based on pathological examination (or) the current clinical practice guidelines; (2) association of pretreatment LMR with overall survival (OS), disease free survival (DFS), cancer special survival (CSS), or other survival rates were reported; 3)cox regression; (4) Risk ratio (HR) with 95% confidence interval (CI) can be extracted or calculated from the literature; (5) Patients were divided into high and low LMR groups according to the truncation value; (6) The full text of the article is published; (7) The data collected primarily from distant (M) metastatic colorectal cancer. The exclusion criteria are as follows: (1) Repeatedly published studies or secondary analyses, *in vitro* experiments, animal experiments, reviews, letters, guidelines, case reports, pathological mechanisms, conference abstracts, or systematic reviews; (2) Colorectal cancer patients without metastasis; (3) There is no cut-off value; (4) Overlapping or duplicate data.

### 2.3 Data extraction

Two researchers (PP.M, LL) independently screened all articles identified by the database. First, EndNote X 9.0 software was used to remove duplicate literature, excluding case reports, conference abstracts, letters, and review articles. Then, the titles and abstracts of the remaining literature were screened to exclude studies that were not relevant to the topic, and the full texts and supplementary information of the remaining studies were reviewed to identify eligible studies. Finally, data were extracted according to a unified extraction table, which included information such as first author name, year of publication, country, Study type, sample size, patient age, study period, TNM stage, follow-up time, cut-off value, and HR (95% CI) of Study center, OS, PFS, DFS, and CSS. The two researchers (PP.M,LL) independently cross-checked all included papers and extracted data. Any disputed articles were referred to a third researcher (XT,G) for consensus resolution.

### 2.4 Quality assessment

The Newcastle-Ottawa Quality Assessment Scale (NOS) was used for independent evaluation in terms of selection, comparability, and outcome (21). A total of 9 items were extracted, and studies achieving scores of 1 as well as 7-9 in each item were considered to be of high quality (22).

### 2.5 Statistical analysis

The pooled HR and 95% CI were calculated, and the MLR correlation results were converted into LMR format as necessary to evaluate the prognostic value of LMR in mCRC patients. It should be noted that for studies reporting MLR data (23, 24), we took the reciprocal of the corresponding HR values and confidence intervals, exchanging the upper and lower confidence limits to convert MLR into LMR values for ease of statistical analysis. Heterogeneity was assessed using Cochran’s Q test and Higgins I^2^ statistic. The I^2^ value was used to evaluate the degree of heterogeneity, with I^2^ ≤ 25%, 25%<I^2^<75%, and I^2^≥75% indicating low, moderate, and high levels of heterogeneity respectively. Subsequently, a random effects model was employed for data analysis when I^2^ exceeded 50%; otherwise, a fixed effects model was applied. Subgroup analysis and sensitivity analysis were conducted to validate the robustness of OS and PFS results. Funnel plots as well as Egger’s and Begg’s tests were performed to assess potential publication bias. A significance threshold of P<0.05 was adopted for evaluating statistical significance. Statistical analyses were conducted using STATA 15.0 software and Review Manager 5.

## 3 Results

### 3.1 Study characteristics

**Figure 1:**
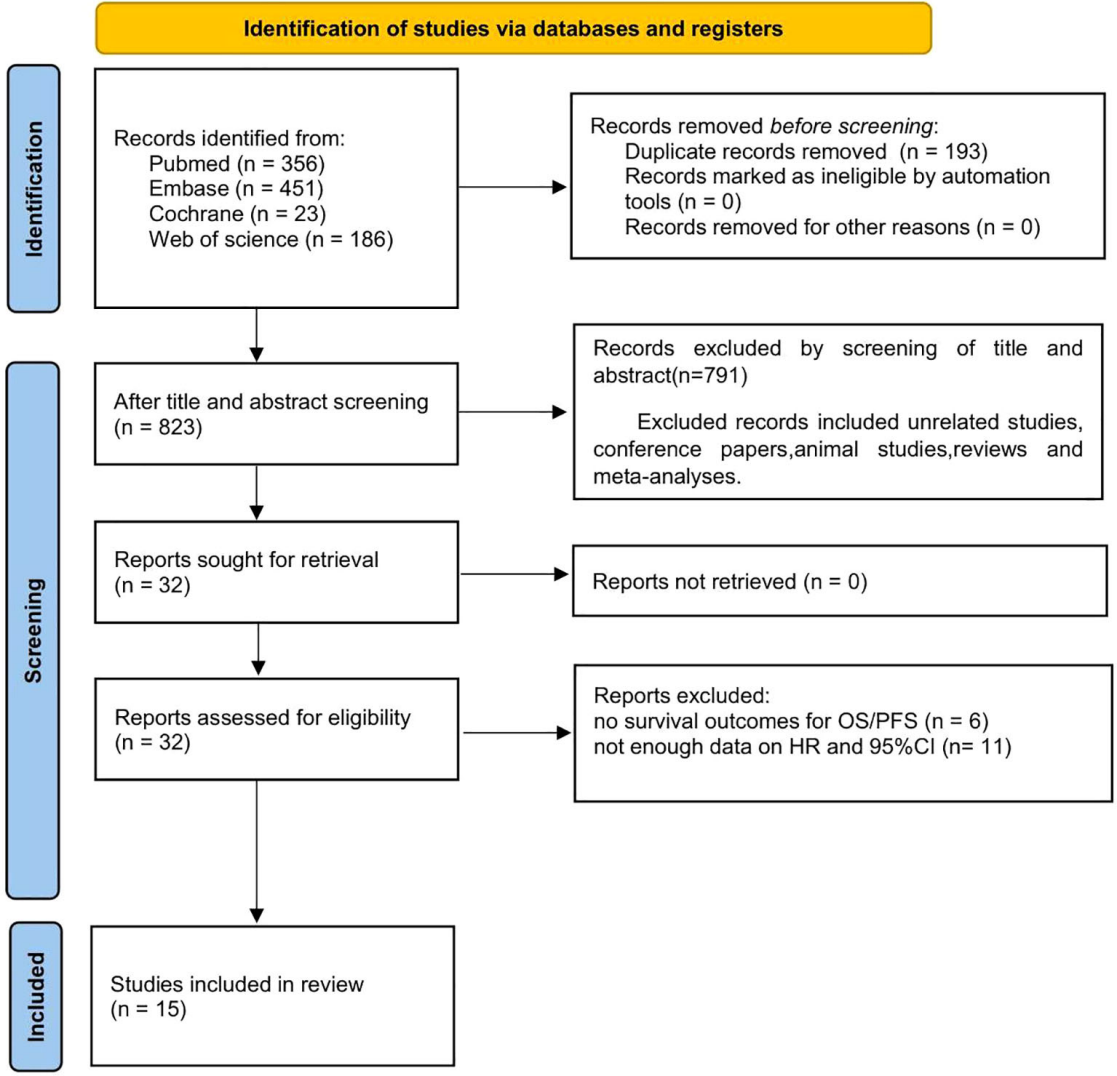
Flow chart of literature screening.

Employing the search strategy mentioned above, an initial literature search yielded 1016 records. However, after removing duplicates, only 823 studies remained eligible for inclusion. Further screening based on title and abstract resulted in the exclusion of 791 records, leaving a final selection of 32 studies for comprehensive evaluation. Among these, seventeen additional studies were excluded primarily due to insufficient availability of relevant data required for survival information computation. Consequently, this meta-analysis includes fourteen selected publications spanning from years 2015 to 2023 and involving a collective cohort comprising a total sample size of n=3199 patients (13, 23–36) (**Figure 1**).

Eligible studies had the following baseline characteristics. Among the included articles, 14 examined the prognostic effect of LMR on OS, 3 examined DFS, 3 examined CSS, and 3 examined PFS. Eight studies were conducted in Asia and six in Europe. All studies were cohort studies, with 13 articles being retrospective and 2 being prospective. All cohort studies were published in English. All included studies consisted of two groups: high and low LMR. The study characteristics, patient baseline data, and study results of the included studies are listed in **Table 1**.

**Table 1:** Basic characteristics of the included literature.

Author	Year	Sources of patients	Study design	sample size	Age	Duration	TNM stage	Study center	Treatment	Follow-up (median)	LMR cut-off	Outcome	NOS score
Shibutani et al. (13)	2015	Japan	retrospective	104	64	2005-2010	IV	Single center	C	NA	3.38	OS	6
Basile et al. (23)	2020	Italy	retrospective	528	NA	2009-2018	IV	Multicenter	C	55	2.04	OS	8
Li et al. (24)	2022	China	retrospective	196	NA	2010-2015	IV	Single center	S	NA	3.27	OS,PFS	6
Facciorusso et al. (25)	2016	Italy	retrospective	127	66	2003-2012	IV	Single center	RFA	63	3.96	OS	7
Kuramochi et al. (26)	2021	Japan	prospective	32	67	2016-2017	IV	Multicenter	C	NA	3.18	OS,PFS	8
Lin et al. (27)	2016	China	retrospective	488	54	2005-2013	IV	Single center	C	23.5	3.11	OS,PFS	7
Lisanti et al. (28)	2020	Italy	retrospective	168	NA	2005-2018	IV	Multicenter	C	51.5	2.22	OS	7
Neal et al. (29)	2015	UK	prospective	312	66	2006-2010	IV	Single center	S	29.7	2.35	OS,CSS	8
Neofytou et al. (30)	2015	UK	retrospective	140	NA	2005-2012	IV	Single center	Neoadjuvant + S	33	3	OS,CSS,DFS	7
Ouyang et al. (31)	2023	China	retrospective	110	59	2019-2022	IV	Single center	I\I+C\I+T\I+T+C	NA	1.49	OS	7
Ozawa et al. (32)	2015	Japan	retrospective	117	63	1997-2012	IV	Multicenter	C+S	39	3	CSS,DFS	8
Peng et al. (33)	2017	China	retrospective	150	NA	2000 -2012	IV	Single center	C/S/RFA	36	2.82	OS	8
Song et al. (34)	2015	Korea	retrospective	177	52	2006-2013	IV	Single center	S+C+R	3.1	3.4	OS	7
Wang et al. (35)	2019	China	retrospective	452	57	2002-2016	IV	Single center	S+/R/C/RFA	28	3.4	OS,DFS	8
Zager et al. (36)	2020	Israel	retrospective	98	59.2	2009-2018	IV	Single center	S+C	NA	4.4	OS	6

NOS, Newcastle–Ottawa scale; OS, overall survival; PFS, progression-free survival; DFS, disease free survival; CSS, cancer special survival; LMR, lymphocyte/monocyte ratio; S, surgery; C, chemotherapy; R, radiotherapy; RFA, radiofrequency ablation; I, immunotherapy; T, targeted therapy; NA, not available.

### 3.2 The quality assessment of the included studies

The methodological quality of the 15 included studies was assessed using the Newcastle-Ottawa scale (NOS), a well-established tool for evaluating research rigor. Scores on this scale, which range from 5 to 8 stars, are presented in **Supplementary Table S2**.

### 3.3 Meta-analysis results

#### 3.3.1 The effect of LMR on OS

**Figure 2:**
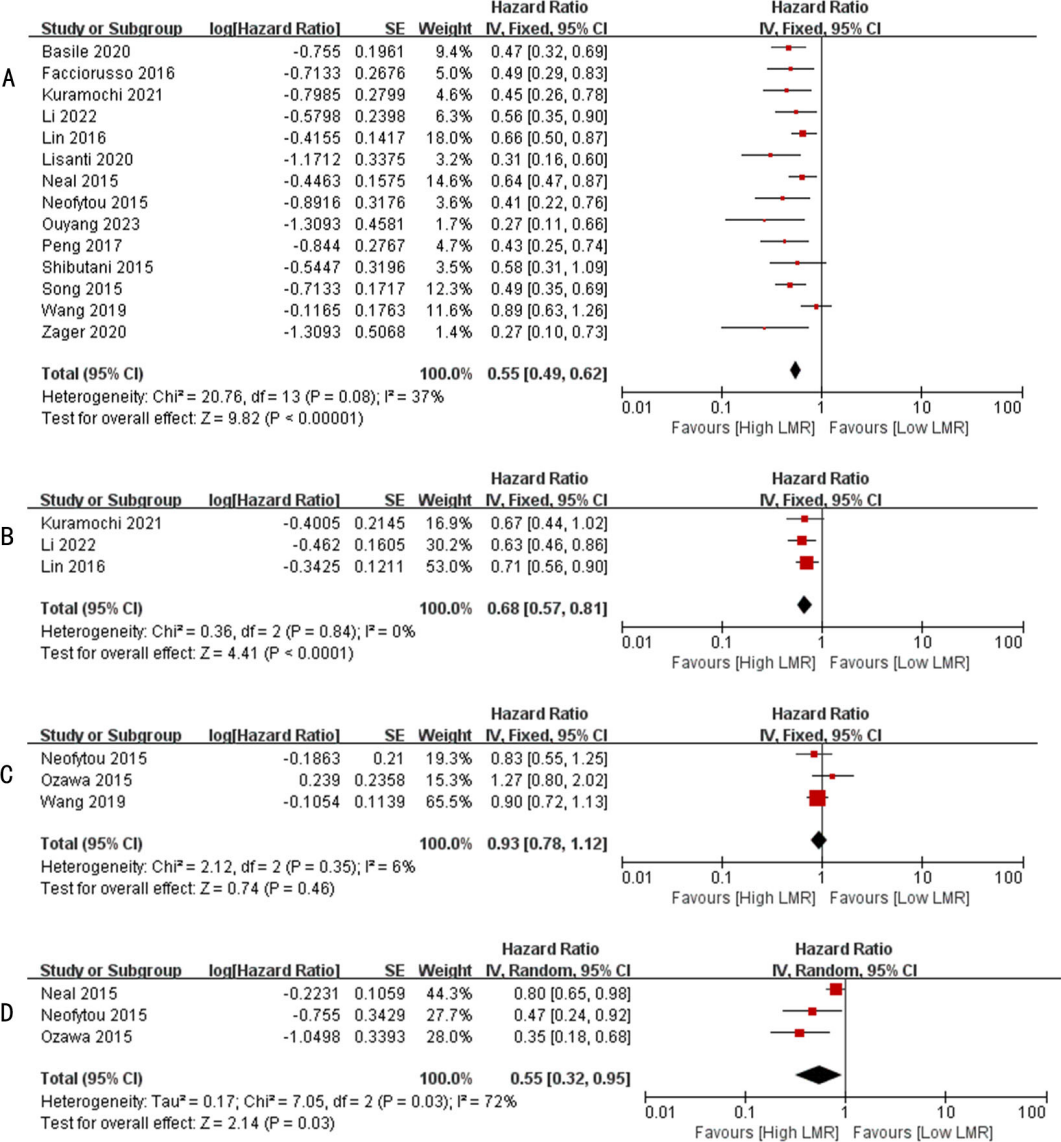
**(A)** Forest plots for the association between LMR and OS; **(B)** Forest plots for the association between LMR and PFS; **(C)** Forest plots for the association between LMR and DFS; **(D)** Forest plots for the association between LMR and CSS.

A total of 14 studies have reported the relationship between LMR levels and OS in patients with metastatic colorectal cancer (13, 23–31, 33–36), involving a total of 3082 patients. Heterogeneity test found no significant heterogeneity among the studies (I^2^ = 37%, p=0.008). Therefore, we performed a meta-analysis using a fixed effects model. Pooled analysis showed that patients with metastatic colorectal cancer with high LMR values were significantly associated with better OS (HR: 0.55, 95%CI: 0.49-0.62, p < 0.00001, **Figure 2A**).

#### 3.3.2 The effect of LMR on PFS

A total of 3 studies have reported the relationship between LMR level and PFS in patients with metastatic colorectal cancer (24, 26, 27), involving a total of 716 patients. Heterogeneity test found no significant heterogeneity among the studies (I^2^ = 0%, p=0.84). Therefore, we performed a meta-analysis using a fixed effects model. Pooled analysis showed that patients with metastatic colorectal cancer with high LMR values were significantly associated with better PFS (HR: 0.68, 95%CI: 0.57-0.81, p < 0.0001, **Figure 2B**).

#### 3.3.3 The effect of LMR on DFS

A total of 3 studies have reported the relationship between LMR levels and DFS in patients with metastatic colorectal cancer (30, 32, 35), involving a total of 709 patients. Heterogeneity test found significant heterogeneity among studies (I^2^ = 6%, p=0.35). Therefore, we performed a meta-analysis using a fixed effects model. Pooled analysis showed no significant association between patients with metastatic colorectal cancer with high LMR values and DFS (HR: 0.93, 95%CI: 0.78-1.12, p=0.46, **Figure 2C**).

#### 3.3.4 The effect of LMR on CSS

A total of 3 studies have reported the relationship between LMR levels and CSS in patients with metastatic colorectal cancer (29, 30, 32), involving a total of 569 patients. Heterogeneity test found no significant heterogeneity among the studies (I^2^ = 72%, p=0.03). Therefore, we performed a meta-analysis using a random effects model. Pooled analysis showed that patients with metastatic colorectal cancer with high LMR values were significantly associated with better CSS (HR: 0.55, 95%CI: 0.32-0.95, p=0.03, **Figure 2D**).

### 3.4 Subgroup analysis

To detect potential heterogeneity, we performed subgroup analyses based on sample size, cutoff values, study centers, year of publication, and region. **Table 2** shows the results of these analyses. Our findings suggest that patients with high LMR had better OS regardless of study center (single or multi-center), region of publication (Asia or Europe), year of publication (2015-2017 or 2018-2023), sample size (≤150 or >150), and cutoff (≤3 or >3). However, in the articles published from 2018 to 2023, the heterogeneity test found significant heterogeneity across studies (I^2^ = 62%).

**Table 2:** Pooled HRs for OS in subgroup analyses.

Subgroup	Number of Studies	Patient	HR [95%CI]	*P* value		*I* ^2^
***Total***	14	3082	0.55 [0.49-0.62]	<0.00001	37%	
***Sample size***						
≤150	7	761	0.44 [0.34-0.55]	<0.00001	0%	
>150	7	2321	0.60 [0.52-0.69]	<0.00001	49%	
***LMR cut-off***						
LMR≤3	6	1408	0.49 [0.40-0.59]	<0.00001	28%	
LMR>3	8	1674	0.60 [0.52-0.69]	<0.00001	37%	
***Study center***						
Multicenter	3	728	0.43 [0.32-0.57]	<0.00001	0%	
Single center	11	2354	0.57 [0.50-0.64]	<0.00001	37%	
***Publish year***						
2015-2017	7	1498	0.56 [0.48-0.65]	<0.00001	0%	
2018-2023	7	1584	0.54 [0.45-0.65]	<0.00001	62%	
***Region***						
Asia	8	1709	0.59 [0.51-0.69]	<0.00001	44%	
Europe	6	1373	0.50 [0.41-0.60]	<0.00001	22%	

R, hazard ratio; CI, confidence Interval; OS, overall survival; LMR, lymphocyte/monocyte ratio.

### 3.5 Correlation between LMR and clinicopathological parameters

Information on LMR and tumor size in mCRC patients was retrieved from four cohorts, which were summarized (OR=1.75, 95%CI: 1.17-2.61, P=0.007, **Supplementary Figure S1A**), indicated that patients with elevated LMR have smaller tumor size. Four studies reported an association between high LMR and carcinoembryonic antigen, however, the combined data did not show such an association (OR=1.52, 95%CI: 0.83-2.78, P=0.18, **Supplementary Figure S1B**). Three studies reported the time to metastasis of LMR in mCRC patients, the combined OR of 1.36 (95% Cl: 0.89-2.08, P =0.16, **Supplementary Figure S1C**) suggested that elevated LMR was not associated with time to metastasis for these patients. In four studies evaluating LMR and the level of tumor differentiation in CRC patients, the results showed no correlation (OR=0.50, 95% CI: 0.24-1.04, P =0.06, **Supplementary Figure S1D**) (see **Table 3**).

**Table 3:** Meta-analysis results of the correlation between LMR and clinicopathological features in patients with mCRC.

	Number of Studies	Meta-analysis results		Effect model	Meta-analysis results	
		OR	P		I^2^ (%)	P
***Primary tumor size***	4	1.75 [1.17-2.61]	0.007	fix-effects model	0%	0.58
***CEA***	4	1.52 [0.83-2.78]	0.18	fix-effects model	0%	0.44
***Time to metastasis***	3	1.36 [0.89-2.08]	0.16	fix-effects model	0%	0.42
***Tumor differentiation***	4	0.50 [0.24-1.04]	0.06	Random-effects model	71%	0.02

primary tumor size, > 5 cm or < 5 cm; CEA, > CEA cut-off vs < CEA cut-off; time to metastasis, Synchronous transfer vs Metachronous transfer; Tumor differentiation, well or moderate vs poor.

LMR, lymphocyte/monocyte ratio; mCRC, metastatic colorectal cancer; OR, odd ratio; CEA, Carcinoembryonic Antigen.

### 3.6 Sensitivity analysis

**Figure 3:**
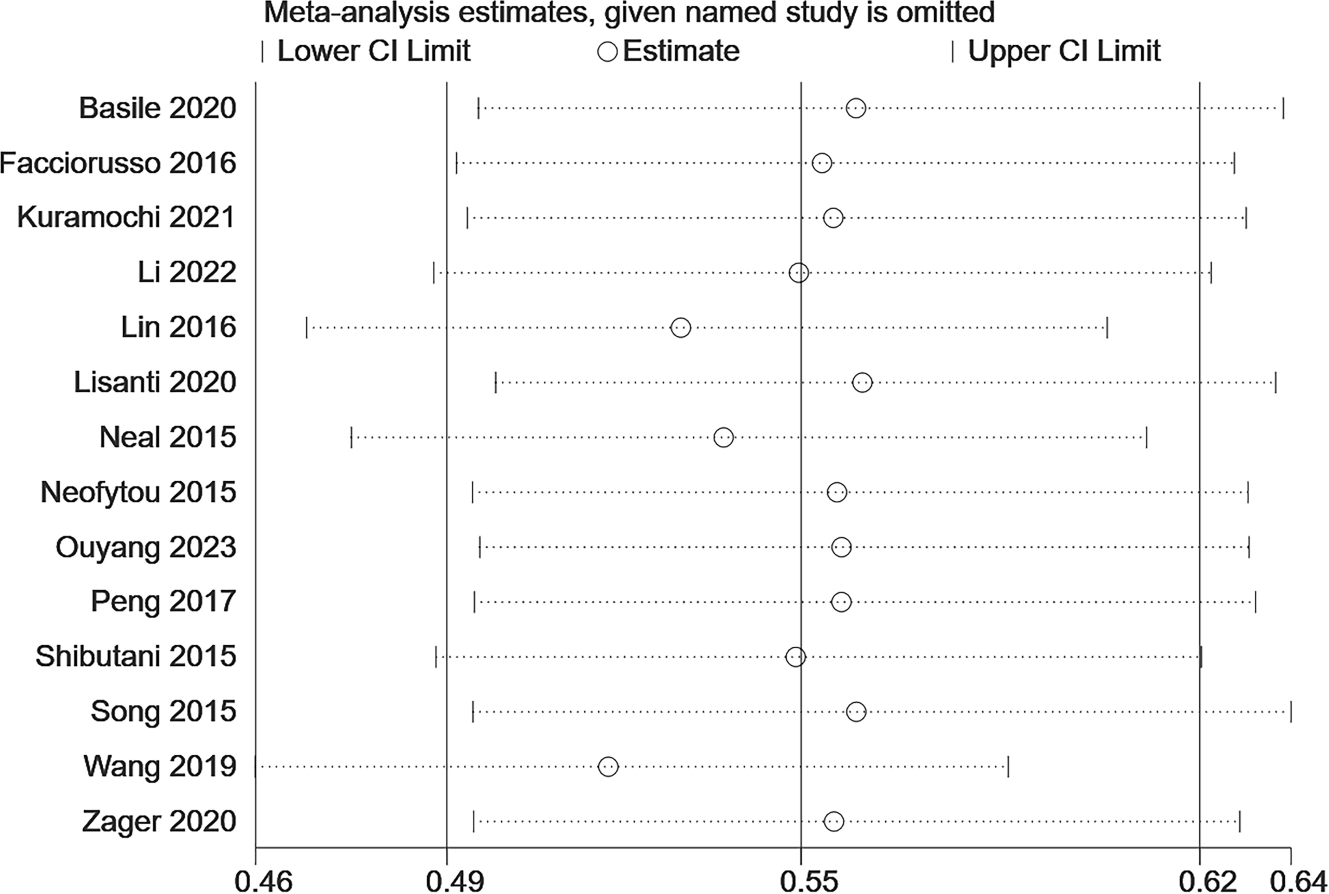
Sensitivity analysis for the association between LMR and OS.

Sensitivity analysis assesses the impact of each study on the overall outcome by removing the included studies one at a time. We conducted a sensitivity analysis on LMR and OS, and found that the effect size of each study after the removal of each study in turn had little change and was still within the original range, which proved that no single study affected the results of OS (**Figure 3**), indicating that the results were relatively credible.

### 3.7 Publication bias

**Figure 4:**
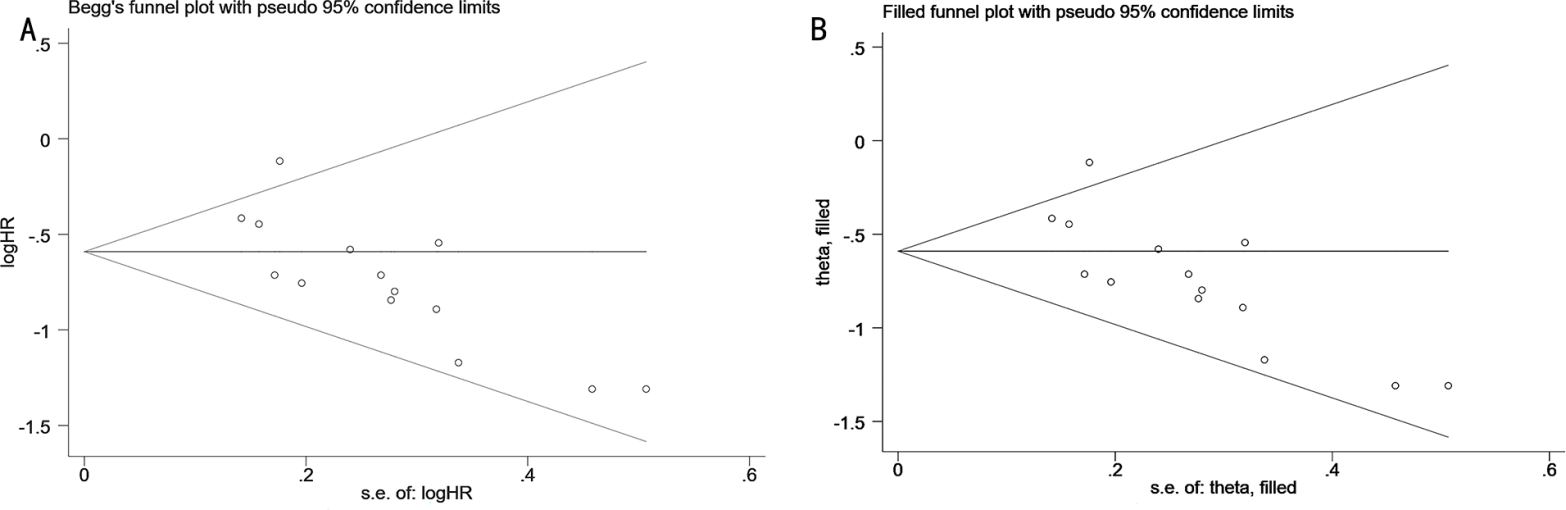
**(A)** Funnel plot for the evaluation of publication bias for the association between LMR and OS; **(B)** Trim-and-fill funnel plot for OS.

Among the above results, there were enough articles (>10 studies) included in the analysis of the relationships between LMR at baseline and OS. To assess publication bias, HR for OS and its associated 95% CI were aggregated and evaluated using funnel plots as well as Begg and Egger tests. The shape of the funnel plot indicates that there is publication bias in the included LMR and OS studies (Egger’s P=0.003 and Begg’s P=0.006; **Figure 4A**). Next, we used trim-and-fill methods to evaluate the symmetry of the funnel chart, by supplementing unpublished research, and the final result showed that the difference in effect sizes before and after the Trim-and-fill method was NS, indicating that publication bias had little effect on the results of the Meta-analysis (**Figure 4B**). However, due to a limited number of meaningfully evaluated studies (<10 studies), no publication bias analysis was performed for others.

## 4 Discussion

Tumor-related inflammation is still an important research area, and a large number of studies have shown its influence on the occurrence and progression of cancer (37). Blood-derived parameters provide an easily accessible and repeatable method for assessing systemic inflammation as an objective biomarker for predicting patient outcomes (38, 39). However, there is evidence that a high lymphocyte-to-monocyte ratio is associated with improved survival outcomes in these patients, and LMR remains a valuable predictor of long-term prognosis (40). In this study, reliable evidence from 15 studies in 3199 cases was comprehensively analyzed to explore the prognostic significance of LMR in mCRC patients. Combined hazard ratios consistently showed that increased LMR was significantly associated with improved OS (HR: 0.55, 95%CI: 0.49-0.62, p < 0.00001). Subgroup analysis was performed to further evaluate the relationship between LMR and OS. Subgroup analysis stratified by truncation value, sample size, study center, region, and publication year was consistent with the combined results. In addition, higher baseline LMR was positively associated with better PFS and CSS, suggesting that higher LMR may signal better outcomes in patients with mCRC. Exceptionally, high LMR values had no significant prognostic value for DFS in patients with metastatic colorectal cancer. Clinicopathological factors in different groups of patients affect their long-term outcomes. We compared four major clinicopathological factors in patients with elevated and normal LMR, and the results were comparable between the two groups. Finally, sensitivity analysis and publication bias assessment were performed, and the results were not statistically significant. Together, our findings suggest that LMR holds promise as a potential peripheral blood biomarker and an effective tool for stratifying patients who may benefit from treatment for mCRC.

In the past few decades, LMR as an indicator of inflammation, which can be measured in plasma or serum, has been more popular, easier to estimate, and more readily available than other indicators of systemic inflammation. Pre-treatment LMR has been shown in many studies to reflect systemic inflammation and is positively associated with the prognosis of various solid tumors, such as melanoma, classical Hodgkin lymphoma, and gastric cancer (41, 42). The lymphocyte and monocyte levels, which serve as indicators of anti-tumor immunity and tumor burden (43), are crucial for the determination of LMR. A decreased count of lymphocytes can impair the immune response against tumor cells, where T lymphocytes play a vital role in recognizing and eliminating these cells, thereby inhibiting tumor cell proliferation and metastasis (44). Tumor-infiltrating lymphocytes (TILs) are pivotal for cell-mediated antitumor immune responses, with increased TILs being associated with improved outcomes in cancer patients. CD4 T cell infiltration triggers the activation of CD8 T cells, promoting apoptosis and cytotoxic activity against cancer cells (45, 46). Studies have shown that reduced lymphocyte counts can lead to reduced survival in various cancers and also diminish the effectiveness of ICIs (27, 47). In addition to lymphocytes, circulating monocytes possess the capacity to differentiate into tumor-associated macrophages (TAMs), which play a pivotal role in the context of tumor-associated inflammation. TAMs exhibit dual functionality by attracting tumor-associated chemokines to recruit peripheral blood monocytes and by modulating innate immune responses and regulatory T cells, thereby exerting suppressive effects on anti-tumor immunity while also promoting angiogenesis and extracellular matrix degradation. Consequently, these effects promote the occurrence and progression of tumors (25). Therefore, monocytes have a crucial function in the tumor immune microenvironment, where TAMs can be identified as high cancer burden markers (48). In this case, the lymphocyte-to-monocyte ratio (LMR) serves as a comprehensive indicator reflecting both the host’s immune status and the extent of tumor development. A decreased LMR, characterized by reduced lymphocyte count and elevated monocyte count, signifies compromised antitumor immunity and increased tumor burden (34). Consequently, LMR holds promise as a more effective prognostic marker for predicting outcomes in patients with mCRC.

To our knowledge, this meta-analysis is the first to comprehensively examine the prognostic significance of LMR in patients with mCRC. Previous meta-analyses of colorectal cancer survival and systemic inflammatory measures have mostly mixed metastatic and non-metastatic colorectal cancer together (16, 18, 19), which may obscure the true relationship between outcomes and prognostic measures, especially given differences in treatment strategies and survival. To the best of our knowledge, only one prior study has conducted a meta-analysis on the prognostic significance of platelet-to-lymphocyte ratio (PLR) in patients with metastatic colorectal cancer (49). However, this meta-analysis represents the first comprehensive evaluation of the prognostic value of LMR in patients with metastatic colorectal cancer. Our study included a large number of qualified, high-quality studies, further confirming that elevated LMR is associated with good OS. These findings are consistent with previous studies of LMR and prognosis in other tumor types. In addition, the subgroup analysis was consistent with the combined results. However, this conclusion may have been influenced by the small number of studies included. Therefore, the prognostic value of LMR needs to be further validated through large-scale and rigorously designed studies.

Although this study provides more detailed outcome measures that enhance the reliability of the conclusions and provide more robust evidence-based medical insights, our meta-analysis has some limitations. First, all eligible studies were from Asia and Europe, mostly from China and Japan, which limits generalizability in regions such as Africa or the Americas. Second, we do not impose restrictions on nationality or region in selecting eligible studies. Only English-language publications were considered. Therefore, it remains necessary to validate the prognostic effect of LMR in mCRC patients treated worldwide. Third, the majority of the included studies were retrospective in nature and had limited sample sizes. Only three studies assessed the significance of LMR in predicting PFS, which may not provide sufficient evidence to establish robust findings. Additionally, there was insufficient data available to support the consistency of outcomes between LMR and DFS as well as CSS. Fourth, the studies included in the analysis had varying LMR cutoff values, ranging from 1.49 to 4.4. To avoid the inherent heterogeneity caused by data inconsistencies in this meta-analysis, a standard cutoff value for LMR is required for future studies. Fifth, because the treatment protocols used in each trial are not uniform, such as radiotherapy and chemotherapy alone, radiotherapy and chemotherapy combined with biological inhibitors, etc., which may affect the reliability of the conclusion. In the future, the effect of the same treatment regimen on the relationship between LMR and prognosis needs to be further discussed.

## 5 Conclusion

In conclusion, this study shows that high levels of LMR are strongly associated with survival outcomes in patients with metastatic colorectal cancer, as well as favorable outcomes for OS, CSS, and PFS. The present findings underscore the capacity of LMR to serve as an independent prognostic biomarker in patients diagnosed with mCRC, thereby offering valuable insights for treatment decision-making. In the future, the clinical value of these biomarkers still needs to be further evaluated in prospective, large-scale, and multicenter studies.

## Data Availability

The original contributions presented in the study are included in the article/**Supplementary Material**. Further inquiries can be directed to the corresponding authors.

## Supplementary material

Supplementary Figure 1
**(A)** Forest plots for the association between LMR and primary tumor size; **(B)** Forest plots for the association between LMR and CEA; **(C)** Forest plots for the association between LMR and time to metastasis; **(D)** Forest plots for the association between LMR and Tumor differentiation.
