# Supplementary figures and images for: The prognostic utility of the ratio of lymphocyte to monocyte in patients with metastatic colorectal cancer: a systematic review and meta-analysis
Source: Front Oncol. 2025 Feb 3;15:1394154. doi: 10.3389/fonc.2025.1394154 (PMC11830611; doi:10.3389/fonc.2025.1394154)

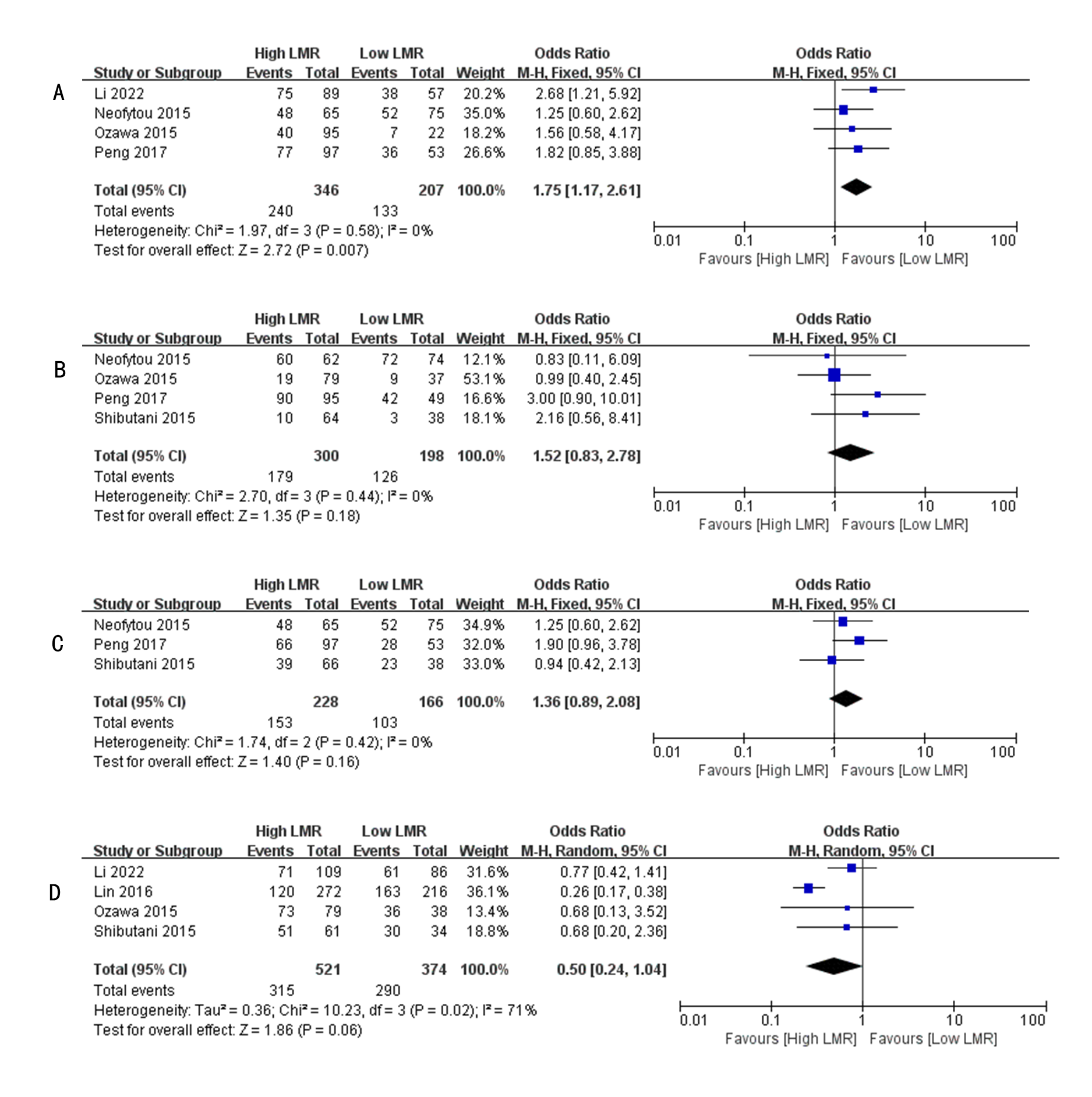

Supplement: Supplementary Figure 1 — (A) Forest plots for the association between LMR and primary tumor size; (B) Forest plots for the association between LMR and CEA; (C) Forest plots for the association between LMR and time to metastasis; (D) Forest plots for the association between LMR and Tumor differentiation. [file Image1.tif]
