# Supplementary material for: The prognostic utility of the ratio of lymphocyte to monocyte in patients with metastatic colorectal cancer: a systematic review and meta-analysis
Source: Front Oncol. 2025 Feb 3;15:1394154. doi: 10.3389/fonc.2025.1394154 (PMC11830611; doi:10.3389/fonc.2025.1394154)
Supplement: Supplementary file 2 [file DataSheet1.zip › Supplementary Table 1.DOCX]

| Supplementary Table S1. Detailed search strategy in four databases. | |
| --- | --- |
| Database | Search strategy |
| Pubmed | (((Lymphocytes) AND (Monocytes)) AND (ratio))) AND(Colorectal Neoplasm) AND(Neoplasm Metastasis ) |
| Embase^*^ | #1 Colorectal Neoplasm  #2 Neoplasm Metastasis  #3 monocyte lymphocyte ratio  #4 lymphocyte monocyte ratio  #5 #3 or #4  #6 #1 and #2 and #5 |
| Web of Science | #1 Colorectal Neoplasm(Topic) or Intestinal Neoplasms (Topic) or Colon Neoplasm (Topic)  #2 Neoplasm Metastasis (Topic)  #3 monocyte to lymphocyte ratio (Topic) or lymphocyte to monocyte ratio (Topic)  #4 #1 AND #2 AND #3 |
| Chochrane | #1Colorectal Neoplasm  #2Neoplasm Metastasis  #3 (Lymphoid Cells) OR (Cell, Lymphoid) OR (Cells, Lymphoid)  #4(monocytes) OR (monocytes, activated killer)  #5 ratio  #6 #3 and #4 and #5  #7 #1 and #2 and #6 |
