# Supplementary material for: The prognostic utility of the ratio of lymphocyte to monocyte in patients with metastatic colorectal cancer: a systematic review and meta-analysis
Source: Front Oncol. 2025 Feb 3;15:1394154. doi: 10.3389/fonc.2025.1394154 (PMC11830611; doi:10.3389/fonc.2025.1394154)
Supplement: Supplementary file 2 [file DataSheet1.zip › Supplementary Table 2.docx]

| Supplementary Table S2. Quality evaluation of the eligible studies with Newcastle–Ottawa scale. | | | | | | | | | |
| --- | --- | --- | --- | --- | --- | --- | --- | --- | --- |
| Study | Selection | | | | Comparability | | Outcome | | |
|  | Representative-ness | Selection of  non-exposed | Ascertainment  of exposure | Outcome not present at start | Comparability on most important factors | Comparability on other risk factors | Assessment of outcome | Long enough follow-up (median≥1 year) | Adequacy  (completeness) of follow-up |
| Shibutani et al.[13] | * | * | * | * | - | - | * | - | * |
| Basile et al.[23] | * | * | * | * | * | - | * | * | * |
| Li et al.[24] | * | * | * | * | - | - | * | - | * |
| Facciorusso et al.[25] | * | * | * | * | - | - | * | * | * |
| Kuramochi et al.[26] | * | * | * | * | * | - | * | * | * |
| Lin et al.[27] | * | * | * | * | - | - | * | * | * |
| Lisanti et al.[28] | * | * | * | * | - | - | * | * | * |
| Neal et al.[29] | * | * | * | * | * | - | * | * | * |
| Neofytou et al.[30] | * | * | * | * | - | - | * | * | * |
| Ouyang et al.[31] | * | * | * | * | * | - | * | - | * |
| Ozawa et al.[32] | * | * | * | * | * | - | * | * | * |
| Peng et al.[33] | * | * | * | * | * | - | * | * | * |
| Song et al.[34] | * | * | * | * | * | - | * | - | * |
| Wang et al.[35] | * | * | * | * | * | - | * | * | * |
| Zager et al.[36] | * | * | * | * | - | - | * | - | * |
| *indicates criterion met; - indicates significant of criterion not met. | | | | | | | | | |
